# Structurally unique PARP‐1 inhibitors for the treatment of prostate cancer

**DOI:** 10.1002/prp2.586

**Published:** 2020-04-28

**Authors:** Ali Divan, Mukund P. Sibi, Alexei Tulin

**Affiliations:** ^1^ School of Medicine & Health Sciences University of North Dakota Grand Forks ND USA; ^2^ North Dakota State University Fargo ND USA

**Keywords:** PARP‐1, PARP‐1 inhibitors, poly(ADP‐ribose), prostate cancer cells

## Abstract

The prognosis for metastatic castration‐resistant prostate cancer is unfavorable, and although Poly(ADP)‐ribose polymerase‐1 (PARP‐1) inhibitors have shown efficacy in the treatment of androgen‐receptor dependent malignancies, the limited number of options present obstacles for patients that are not responsive to these treatments. Here we utilize an integrated screening strategy that combines cellular screening assays, informatics, in silico computational approaches, and dose‐response testing for reducing a compound library of confirmed PARP‐1 inhibitors. Six hundred and sixty‐four validated PARP‐1 inhibitors were reduced to 9 small molecules with favorable physicochemical/ADME properties, unique chemical fingerprints, high dissimilarity to existing drugs, few off‐target effects, and dose‐responsivity in the 1 µmol/L ‐ 20 µmol/L range. The top 9 unique molecules identified by our integrated screening strategy will be selected for further preclinical development including cytotoxicity testing, effects on mitosis, structure‐activity relationship, physicochemical/ADME studies, and in vivo testing.

**Abbreviations**
AR: androgen receptor
PARP‐1: Poly(ADP)‐ribose polymerase‐1
PC: prostate cancer

## 1 INTRODUCTION

There are 15 FDA‐approved therapies for the treatment of prostate cancer (PC). Although androgen blocking therapies have greatly improved the 5‐year survival rate of prostate cancer patients, the majority of patients eventually develop a castration resistant form of metastatic prostate cancer (mCRPC). Ten of the FDA‐approved PC drugs are known to provide some therapeutic benefit against mCRPC, although the overall prognosis for mCRPC remains unfavorable. Poly (ADP)‐ribose polymerase‐1 (PARP‐1) is critical for maintaining chromatin in an accessible state for transcription of pro‐tumorogenic genes downstream of the PC androgen receptor (AR) signaling cascade.^1^, ^2^ Presently, the majority of PARP‐1 inhibiting cancer therapies are competitive antagonists of NAD+, which is the substrate for PARP‐1. Rucaparib, which has a 3D chemical fingerprint similar to the clinical PARP‐1 inhibitors Olaparib and Velaparib,^3^ is the only PARP‐1 inhibitor that is clinically approved for treatment of mCRPC. The limited number of treatment options for mCRPC and the limited diversity of PARP‐1 inhibitors for the treatment of mCRPC present obstacles for treating patients who are not responsive to existing therapies.

To address the need for identifying new PARP‐1 inhibitors, we previously performed a high‐throughput cell‐free reporter assay of 50 000 + small molecules. To reduce the likelihood of identifying redundant NAD + mimetics or non‐specific inhibitors of PARP‐1, the cell‐free assay was designed to identify small molecules that inhibited the histone H4 binding domain of PARP‐1.^3^ Six hundred and sixty‐five compounds were identified as structurally distinct inhibitors of PARP‐1, all of which had IC50 values below or similar to the currently known PARP‐1 inhibitors. Using 3D chemical fingerprint clustering of the positive hits, a single compound with the least structural similarity to any known PARP‐1 inhibitor was selected. This compound, 5F02, has been tested in vitro and in vivo, and is currently undergoing further preclinical development.^3^, ^4^ Here, we utilize a screening strategy that integrates cellular screening, informatics, computational approaches, and dose‐response testing to reduce the remaining 664 small molecules to the top 9 candidates for further development. Selections are made on the basis of favorable predicted physicochemical/ADME properties, unique chemical fingerprints, dissimilarity to existing drugs in development, and fewest off‐target effects.

## 2 EXPERIMENTAL METHODS

### 2.1 Cell culture and migration assay

Ninety‐six‐well plates with silicone stoppers were purchased from Playtpus Technologies (CMA5.101). PC3 cell lines were purchased from ATCC and cultured in 96‐well plates in 10% RPMI (Gibco A1049101) supplemented with non‐essential amino acids (Gibco 11‐140‐050) and penicillin/streptomycin (Gibco 10378‐016). Cell lines were confirmed as mycoplasma negative using Lonza Mycoalert mycoplasma detection kit (LT07‐118). Each well was seeded with 1 × 10^5^ cells (passage number < 8) and cultured for 48 hours. Silicone stoppers were removed, and entire wells were imaged immediately on Biotek Cytation3 imaging system (t = 0). Medium was removed from each well by vacuum and all wells were replenished with serum‐free RPMI supplemented with 6.5 µmol/L test inhibitors, 0.13% DMSO, 10 µmol/L Olaparib (Adooq A10111‐10), or 50 µmol/L Rucaparib (Adooq A10045‐5). Twenty‐four hours after stopper removal samples were imaged again. Each plate was run in triplicate using a scheme of one unique sample per well, and 3 plates per run. Data were analyzed using ImageJ MRI Wound Healing Tool plugin. All area measurement data were normalized to t = 0 control values. The cutoff criteria for positive hits was µ_s_−σ_s_ ≥ µ_v_ + 3σ_v_, where µ is mean, s is sample, σ is standard deviation, and v is vehicle. Statistics were performed with GraphPad Prism software on samples meeting the cutoff criteria using one‐way ANOVA, with FDR = 0.05. Multiple comparisons were corrected for using the two‐stage linear step‐up procedure of Benjamini, Krieger and Yekutieli.

### 2.2 Chemical purity

Experimental inhibitors tested were obtained from the ChemDiv Representative Diversity Set, comprising 50 000 molecules. Samples were validated by ChemDiv ^1^H‐NMR and HPLC/LCMS. All compounds met a minimum requirement of ≥95% purity, and elemental analysis revealed carbon, hydrogen, and nitrogen values were within 0.4% of expected values.

### 2.3 Cell viability assay

Cells were seeded and cultured in 96‐well plates for 48 hours, as described above. Wells were treated for 24 hours in quadruplicate with no stimulus (NT), 0.13% DMSO (veh), 20uM Olaparib (Adooq A10111‐10), 50uM Rucaparib (Adooq A10045‐5), or 70% ethanol. Media were removed from each sample and replaced with PBS containing 0.5 ug propidium iodide (Thermo P1304MP) and 1uL Oligreen (Thermo O7582). Samples were imaged on Biotek Cytation3 using GFP and RFP filter sets. Oligreen fluorescence was used to quantify all cells, and propidium iodide fluorescence was used to quantify dead cells. Cells were counted using Trainable Weka Segmentation plugin for ImageJ/FIJI (Arganda‐Careeras 2017). %Live cells were computed as 100*(All cells – Dead cells).

### 2.4 Chemical taxonomy

Small molecule taxonomy was determined using Classyfire application.^5^ Molecules were queried using SMILES IDs, and direct parent data were reported for each sample.

### 2.5 SWISS‐ADME screening

Samples were input into SWISS‐ADME system using SMILES IDs and all data output were saved in .csv format. Samples meeting the following criteria were considered positive hits for favorable physicochemical properties: 20 < TPSA <130; −0.7 < (xLogP3/wLogP)/2 < 5; 0 < [‐Log(SILICOS‐IT)]‐1.2 < 6; 150 < MW <500; 0.25 < Fraction Csp3 < 1; 0 < rotatable bonds < 9. Samples having the following PAINS alerts were removed: ene_six_het_A, hzone_phenol_B, anil_di_alk_A, quinone_A, azo_A, imine_one_A, mannich_A, anil_di_alk_B, anil_di_alk_C, ene_rhod_A, hzone_phenol_A, ene_five_het_A, anil_di_alk_E. All samples flagged as Brenk alerts were removed, except for those flagged as quaternary nitrogens. Samples with low predicted intestinal absorption, synthetic accessibility > 3.5, or > 2 CyP enzymes inhibited were also excluded.

### 2.6 2D and 3D chemical fingerprinting

For 2D fingerprinting, samples were input into the ChemMine database using SMILES format. Single linkage distance matrix hierarchical clustering was performed, selecting Z‐scores for the display value. Data were exported into .csv format. 3D chemical fingerprinting was performed using Canvas 1.6 software. Molecules were clustered into a 10 × 10 matrix based on self‐organizing maps calculated as the sum of fingerprint distances for all 665 positive hits from the cell‐free assay, as described previously.^3^ All raw data were imported into GraphPad Prism software for heatmap visualization.

### 2.7 Drug similarity

To determine if any of our top hits were in clinical use, compounds were input into the DrugBank 5.0 database by SMILES ID. To determine the phase of research for each compound and all similar compounds, the ChemMine EI search algorithm was used to interrogate the ChEMBL database for molecules with a similarity cutoff of ≥ 0.85. The table view was used to determine the max phase of each molecule.

### 2.8 Multiple Targets

Molecules were entered into the ChEMBL database by SMILES ID and the heatmap view was used to visualize targets of molecules for which data were available. Data were exported to .csv format, pChEMBL activity values were made binary, and data were imported into Graphpad Prism for heatmap visualization.

## 3 RESULTS

### 3.1 Inhibitors of cellular migration

**Figure 1:**
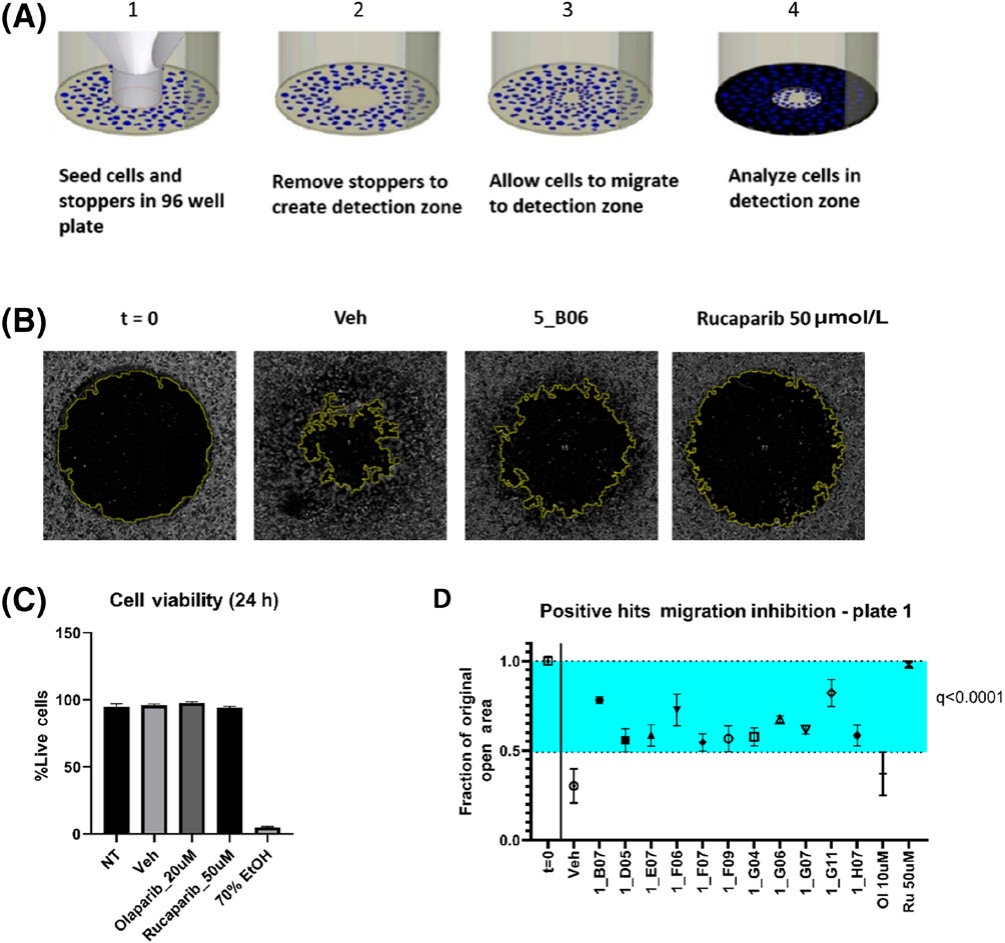
PARP‐1 inhibitors with phenotypic effects in cellular assays. (A) Principle of migration assay in which cells are imaged immediately after stopper removal (step 2) and again after 24 h of culture in serum‐free medium (step 4). (B) Example of images of areas of interest immediately after stopper removal (t = 0), and after 24 h of culture for samples treated with 0.13% DMSO (Veh), 6.5 µmol/L experimental inhibitors (in this case 5_B06), or 50 µmol/L Rucaparib; yellow outline denotes outer boundary of computed open area using ImageJ MRI Wound Healing Tool plugin. (C) Propidium iodide and Oligreen viability assay performed on cells receiving no treatment (NT), 0.13% DMSO (Veh), 20 µmol/L Olaparib, 50 µmol/L Rucaparib, or 70% ethanol. (D) Example of positive hits identified from experimental plate 1 of 8 using µ_s_−σ_s_ ≥ µ_v_ + 3σ_v_ criteria; blue shaded area highlights region considered positive hits; Statistics performed using one‐way ANOVA, FDR = 0.05; multiple comparisons were corrected for using the two‐stage linear step‐up procedure of Benjamini, Krieger and Yekutieli

Six hundred and sixty‐four compounds having cell‐free inhibitory activity on PARP‐1 were tested in vitro using a high‐throughput cellular migration assay developed by Platypus Technologies.^6^ This assay served to evaluate whether any compound could reduce metastatic potential of prostate cancer cells (Figure 1A). Each inhibitor was tested on PC3 cell lines at 6.5 µmol/L concentration and open area was evaluated immediately after the removal of silicone stoppers, and again 24 hours later. Area measurements were calculated using the MRI Wound Healing Tool plugin for ImageJ (Figure 1B). The rationale for using PC3 cell lines was that they are of neuroendocrine origin and are resistant to androgen blocking therapies, which is the typical context in which PARP‐1 inhibitors are used.^7^, ^8^, ^9^ Using 0.13% DMSO as vehicle, 10 uM Olaparib as a weak inhibitor, and 50 µmol/L Rucaparib as a strong inhibitor, the z‐factor of the assay was computed as > 0.6, an ideal value for high‐throughput screening.^10^ Neither Olaparib nor Rucaparib affected cell viability at the time points tested (Figure 1C). For each plate screened, molecules were considered positive hits if the mean of the test sample minus its standard deviation was greater than or equal to the mean of the vehicle minus three standard deviations of the vehicle. Statistical testing was performed on hits meeting the above criteria, and hits with q value < 0.05 were selected for further screening (Figure 1D). This screening method reduced our library of 664 hits to 66 small molecules exhibiting cell migration inhibiting activity. The distribution surface of positive hits was plotted,^11^ and demonstrated that there was no systematic error in hit distribution (Figure S1).

### 3.2 Favorable physicochemical and ADME properties

**Figure 2:**
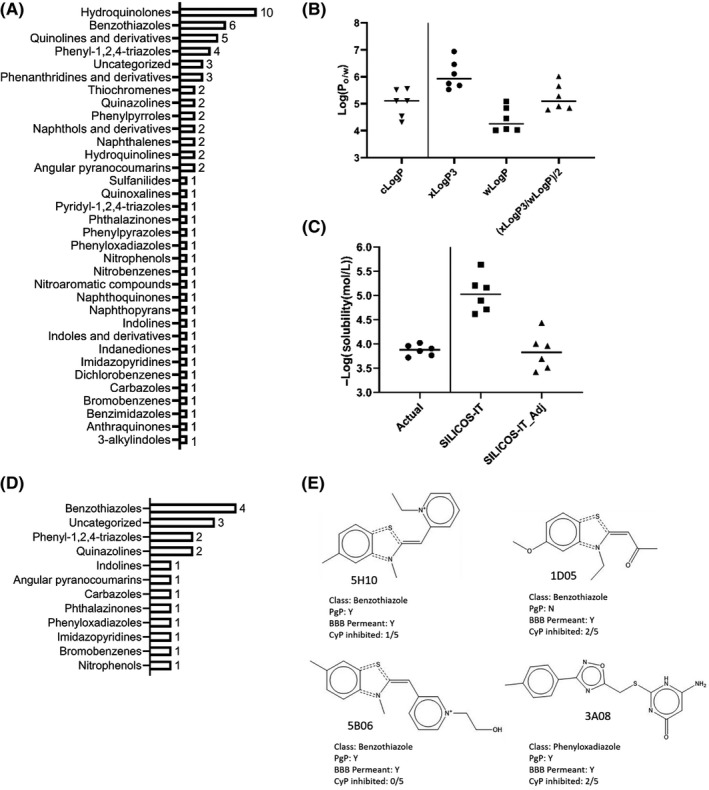
Top hits using SWISS‐ADME application for prediction of physicochemical, PK, ADME, and medicinal properties. (A) Chemical diversity of top 66 compounds identified by cellular migration assay, prior to in silico screening by SWISS‐ADME. (B) Comparison of cLogP values obtained empirically for compounds 5F02, FC‐7220, MC270016, MC270017, MC270019, MC270021 (^4^), and SWISS‐ADME predicted values using xLogP3, wLogP, or the average of both predicted values. (C) Comparison of solubility values (‐Log(S)) obtained empirically for the compounds described in (B), compared to solubility values predicted by the SWISS‐ADME SILICOS‐IT method, and adjustment of those values (SILICOS‐IT_Adj). (D) Chemical diversity of top 19 non‐PAINS, non‐toxic compounds, meeting adjusted SWISS‐ADME cutoff for favorable physicochemical properties. (E) Chemical structures of top 4 molecules predicted to have optimal physicochemical properties, high gastrointestinal absorption, and inhibitory potential against 2 or fewer CyP enzymes; CyP is cytochrome P oxidase; Pgp is P‐glycoprotein efflux pump; BBB is blood–brain barrier

The chemical diversity of the top 66 hits was determined by submitting all compounds into a chemical taxonomic application, which clusters chemicals on the basis of a classification system called ChemOnt.^5^ The 66 compounds clustered into 34 direct parent groups, of which hydroquinolones benzothiazoles, quinolone derivatives, and phenyl‐1,2,4‐triazoles were overrepresented (Figure 2A). To determine whether any of the top 66 small molecules had favorable physicochemical, ADME, or medicinal properties for in vivo use and preclinical development, we utilized SWISS‐ADME, a cheminformatics database for predicting small molecule properties.^12^ With respect to lipophilicity and water solubility, the database provides values using several different algorithms. To determine which algorithm was most appropriate for our use, we queried compounds in which we had previously performed wet physicochemical studies^4^ and compared the predicted results to the actual values. Lipophilicity (cLogP) was most closely approximated by averaging the output of two computational algorithms, xLogP3 and wLogP (Figure 2B).^13^, ^14^ For water solubility, the default parameter used by SWISS‐ADME is the ESOL method.^15^ The SILICOS‐IT method provided the closest approximation to our empirical values.^12^ A minor adjustment to the SILICOS‐IT output further improved the prediction accuracy (See methods for details) (Figure 2C). These adjustments were applied and integrated into SWISS‐ADME cutoffs for size, polarity, saturation, and flexibility. Pan assay interference compounds (PAINS)^16^ were excluded based on those known to be most promiscuous,^17^ and putatively toxic compounds were excluded based on existing SWISS‐ADME criteria.^18^ Overall, this approach reduced the list of 66 hits to 19 non‐PAINS and non‐toxic compounds with favorable physicochemical properties, belonging to 12 direct parent groups (Figure 2D). The top 19 compounds were queried for predicted gastrointestinal absorption, blood–brain barrier permeability, cytochrome P oxidase inhibition, synthetic accessibility, and whether they were substrates for P‐glycoprotein efflux pumps. By requiring that all compounds have gastrointestinal absorption, and no more than 2 of 5 cytochrome P oxidases inhibited, the list of 19 positive hits was reduced to 3 benzothiazoles and 1 phenyloxadiazole (Figure 2E).

### 3.3 Unique chemical fingerprints

**Figure 3:**
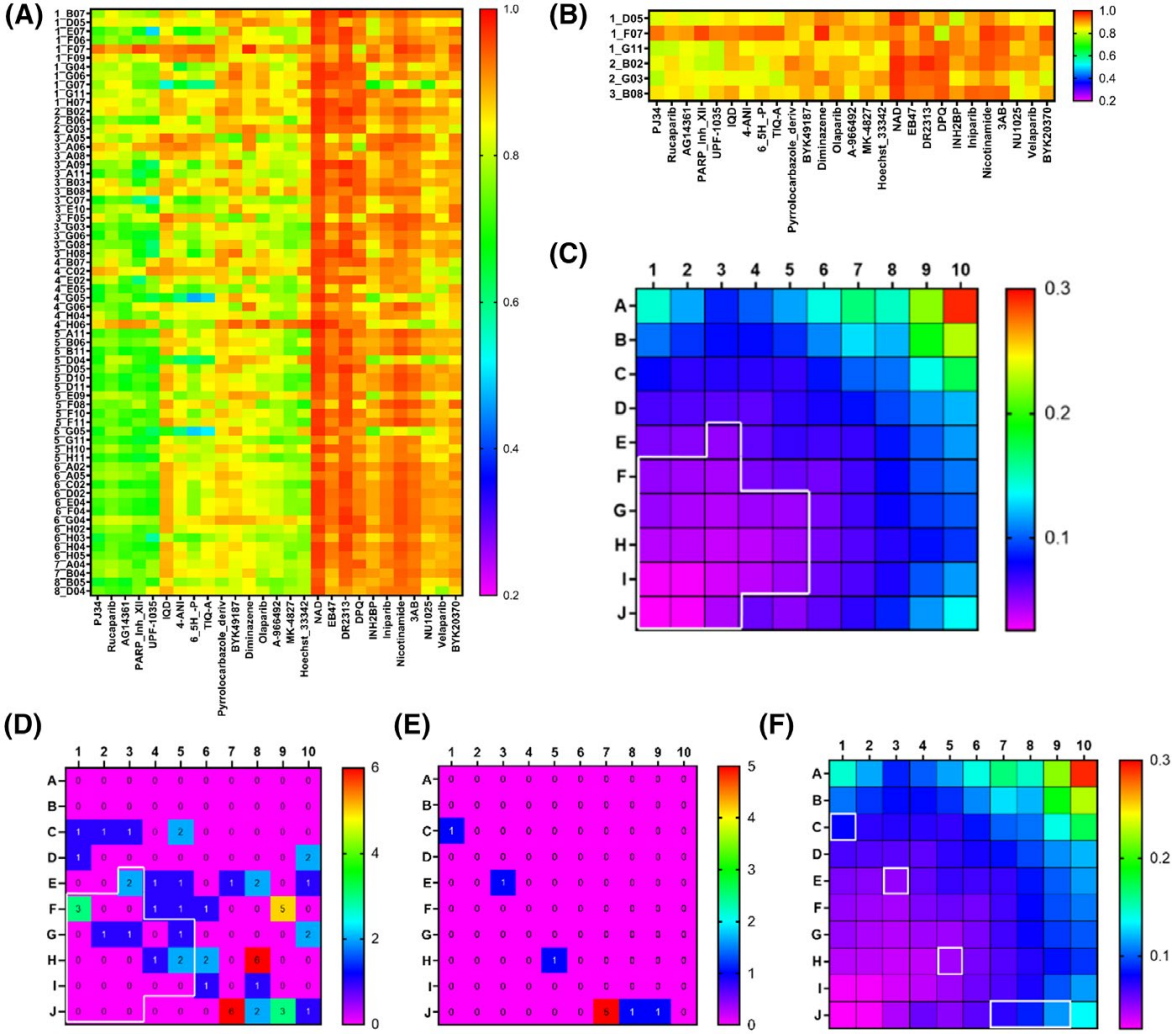
2D/3D chemical fingerprinting and similarity comparisons to known PARP‐1 inhibitors. (A) 2D Single linkage distance matrix hierarchical clustering of top 66 hits compared to 27 known PARP‐1 inhibitors (1 = highly dissimilar, 0 = identical). (B) Selected compounds having dissimilarity score of ≥0.80 from any of the 27 known PARP‐1 inhibitors. (C) Superimposed 3D similarity map comparing all 664 hits from cell free assay to all known PARP‐1 inhibitors; region outlined in white represents area with ≤0.05 Tanimoto similarity to any known PARP‐1 inhibitor. (D) Similarity binning of 66 PARP‐1 inhibitors that were positive hits in cellular migration assay; region outlined in white highlights subset of 11 molecules that have ≤0.05 Tanimoto similarity to any known PARP‐1 inhibitor; (E) 3D binning of molecules identified as positive hits by SWISS‐ADME (2E) or 2D hierarchical clustering (B). (F) Molecules described in (E) (white rectangles), compared to superimposed 3D similarity map of all known PARP‐1 inhibitors

Although taxonomic classification demonstrated high diversity of the top 66 hits, it did not provide any comparisons with existing PARP‐1 inhibitors. To address this shortcoming, we performed single linkage distance matrix hierarchical clustering using ChemMine^19^ and queried our top hits against 27 known PARP‐1 inhibitors (Figure 3A). Values were filtered to include only those compounds that had >0.8 dissimilarity from any known PARP‐1 inhibitors, which reduced the list to 6 compounds (Figure 3B, Figure S2A). We also performed a 3D fingerprint comparison using Canvas 1.6 software which binned similar molecules based on self‐organizing maps calculated as the sum of fingerprint distances for the 66 positive hits from the migration assay (Figure S3). By superimposing the 3D fingerprints of the 27 known PARP‐1 inhibitors, a region of least similarity was identified, wherein each molecule had less than 0.05 Tanimoto similarity to any known PARP‐1 inhibitor (Figure 3C). Eleven compounds, two of which overlapped with hits from the 2D clustering method were identified in the region of least similarity (Figure 3D, Figure S2B). 3D fingerprint binning of the top hits from the 2D clustering, and SWISS‐ADME screening methods demonstrated that all small molecules selected by these methods had less than 0.12 Tanimoto similarity to any known PARP‐1 inhibitor (Figure 3E‐F).

### 3.4 Development landscape

**Figure 4:**
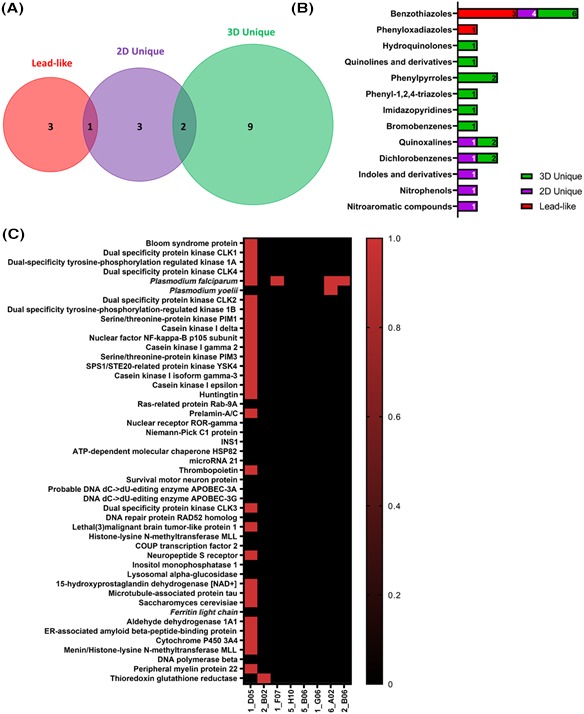
Top hits and off‐target effects. (A) Venn diagram showing number of top hits, and overlapping hits from each selection method; Lead‐like molecules (red) were identified by SWISS‐ADME (Figure 2A‐E); 2D unique molecules (purple) were identified by 2D Single linkage distance matrix hierarchical clustering (Figure 3A,B); 3D unique molecules (green) were identified by 3D self‐organizing maps (Figure 3C‐E); (B) Non‐exclusive representation of direct parent groups of small molecules identified by each selection method. (C) Known non‐PARP‐1 targets for 8 of the 18 compounds shown in (A)

In total, the combined approach of using 3 in silico methods for identifying lead compounds identified 18 molecules belonging to 13 parent groups as candidates. Three of the molecules were hits in two screening methods, and all three screening methods identified benzothiazoles as positive hits (Figure 4A,B, Figure S4). Although the methods employed identified potentially useful molecules for preclinical development, none of those approaches addressed whether any of the molecules were already in clinical use, in early development, or whether they resembled known clinical compounds. To address the first question we queried DrugBank 5.0, an online database containing molecular information about drugs, mechanisms, interactions, and targets in the US, Canada, and EU.^20^ DrugBank did not identify any of our 18 small molecules as currently in clinical use. The structural similarity search function in the ChemMine application was used to determine the status of our compounds with respect to existing development, and whether similar compounds were currently in development. Using a Tanimoto similarity cutoff of ≥0.85, 57 small molecules similar to our top 18 hits were identified. All of the 57 similar molecules were designated as phase 0, meaning that none of them were in preclinical or clinical development (Table S1). Lastly, we queried the ChEMBL database to determine if any of the top 18 hits had known molecular targets other than PARP‐1. Data were available for 8 of the 18 hits. One of the hits (1D05), which was identified as a candidate using the SWISS‐ADME and 2D clustering methods (Figure S4), had reported activity against 29 different targets (Figure 4C). Four other molecules had reported reactivity with thioredoxin glutathione reductase (2B02), *Plasmodium falciparum* (1F07, 6A02, 2B06), or *Plasmodium yoelii* (6A02) (Figure 4C). Given the high number of known non‐PARP1 targets, 1D05 was removed from our list. Lastly, we used the cellular migration assay to perform 5‐point titrations on the top 17 molecules. This assay reduced our top 17 molecules to 9 molecules exhibiting a dose‐response relationship (Figure S5).

## 4 DISCUSSION

The migration assay is an effective screening tool, as it reduced the number of positive hits from 664 to 66. The computed z‐factor was >0.6, which indicates that this approach had high sensitivity and specificity for high‐throughput screening assays.^10^ A possible limitation of our screening approach was that we utilized the neuroendocrine carcinoma‐derived PC3 cell line only and did not include adenocarcinoma prostate cancer cell lines such as LNCaP or DU‐145. Our rationale for using PC3 was that neuroendocrine carcinomas have worse prognoses than adenocarcinomas, and that they are insensitive to androgen ablation, making them appropriate for the clinical context of PARP‐1 inhibitor use.^8^, ^9^ Given the high resource cost of performing multiple high‐throughput screens on several cell lines, the use of PC3 was the most logical choice. Investigators wanting to use our screening strategy would need to carefully consider which cell lines to use for their models of interest.

The SWISS‐ADME database reduced our top 66 hits to 4 molecules that were expected to have favorable physicochemical, ADME, PK, and medicinal properties. Although benzothiazoles were overrepresented in the original list of 66 compounds, it was unexpected that they would represent 3 out of our 4 final hits after SWISS‐ADME prediction algorithms (Figure 2E). All three of the benzothiazoles identified by SWISS‐ADME fell within the same 3D fingerprint bin (Figure 3D, cell j7). An important note on our SWISS‐ADME selection criteria is that we excluded Brenk toxicity alerts concerning the presence of any quaternary nitrogen. The reason for doing so is that our previously identified lead compound, 5F02, is also flagged by a quaternary nitrogen toxicity alert, yet is shown to be safe in vivo.^3^ We also needed to adjust the predicted lipophilicity and solubility values to more closely approximate values that we obtained empirically (Figure 2B,C).^4^ These observations underscore that data produced by in silico screening algorithms must be interpreted with caution, and in the context of the specific application.

2D single linkage distance matrix hierarchical clustering was useful for determining similarities between our top 66 hits and the 27 known PARP‐1 inhibitors that we queried. By selecting a dissimilarity cutoff of >0.8, which approximately correlates to a Tanimoto similarity of <0.2, we quickly reduced the number of candidate molecules from 66 to 6. One of the reasons that we also performed 3D fingerprint binning is because we wanted to determine to what extent dimensionality affected the prediction of similarity. We posited that if dimensionality was not a critical factor, we would find substantial overlap in similarity predictions. Our data demonstrate that although both methods did have *some* overlap in molecules predicted to be structurally unique (Figure 4A), the level of similarity was consistently predicted to be higher using the 2D method. For example, 2D comparison of 1D05 to PJ34 gives a dissimilarity score of 0.82 (similarity of approximately 0.18) (Figure 3A), whereas 3D comparison gives a similarity score of 0.055 (Figure S3, PJ34, cell j7). By including top hits from both approaches, risk is averted against any single method that may have bias.

The combined approach of SWISS‐ADME, 2D single linkage distance matrix hierarchical clustering, and 3D fingerprint binning produced 21 hits, 3 of which were overlapping, narrowing the list from 66 to 18 candidates (Figure 4A,B, Figure S4). Although these candidates represented structurally unique and diverse compounds for testing, we wanted to ensure that we would not be wasting valuable resources synthesizing and testing compounds already in use, in development, or sufficiently similar to drugs in development in which potential intellectual property issues could arise. DrugBank was a useful tool for addressing the question of drugs currently in use, as it queries clinically approved drugs from multiple international agencies comprising the regulatory landscape of the United States, Canada, and the European Union. As expected, none of our top hits were in clinical use, as it would be unusual for approved compounds to be included as experimental compounds in commercial small molecule libraries. ChemMine and ChEMBL addressed the less certain question of whether any investigators were already pursuing our compounds of interest in formalized trials. The similarity search function identified 57 molecules that were similar to our top 18 candidates (Table S1). In all cases, our molecules were unique, not currently being pursued by other entities, nor similar to molecules in development. As a final measure of feasibility, the top 18 molecules were queried in the ChEMBL target database to determine whether any were known to have off‐target effects. Ideally, new or next generation drugs would have fewer side effects than those identified before the age of informatics. Our results demonstrated that only one of our top 18 hits (1D05) had potentially concerning cross‐reactivity with other molecular targets (Figure 4C), reducing our final list of top hits to 17. We chose to consider target activity as binary because the quality of information on activity concentrations was inconsistent. A limitation of this approach is that dosage effects are not taken into account. Thus, this form of decision making would always overestimate the number of potential off‐targets. For the purpose of drug discovery, erring on the side of caution may be the most appropriate course of action. The top 17 molecules were reduced to those that exhibited dose response relationship in the migration assay, which reduced the final list of molecules to 9.

In conclusion, we have utilized an integrated strategy which combines cell‐free, cellular, and in silico assays for reducing a library of 664 small molecules to 9 unique PARP‐1 inhibitors for further development in the treatment of prostate cancer. Future investigations will focus on cytotoxicity testing, effects on mitosis, structure–activity relationship, wet physicochemical studies, and in vivo testing.

## 5 ETHICS STATEMENT

This study was carried out in strict accordance with the recommendations from the Guide for the Care and Use of Laboratory Animals, as provided by the American Association of Accreditation of Laboratory Animal Care (AAALAC).

## DISCLOSURE

AD and AT have patents pending for the use of 5B06, 3A08, 5H10, 1D05, 1F07, 2B02, 1G11, 1H07, 4B07, 7A04, 3A06, 6D02, 6A02, 1G06, 7B04, 2B06, 2G03, and 3B08 as PARP‐1 inhibitors, as described in this manuscript.

## AUTHOR CONTRIBUTIONS

AD designed and executed cellular experiments, designed and executed in silico screening pipeline, interpreted data, and wrote the manuscript. AT selected compound library for screening, provided administrative support, and interpreted data.

## Supporting information

Supplementary Material

Supplementary Material
