# Supplementary material for: Structurally unique PARP‐1 inhibitors for the treatment of prostate cancer
Source: Pharmacol Res Perspect. 2020 Apr 28;8(2):e00586. doi: 10.1002/prp2.586 (PMC7186898; doi:10.1002/prp2.586)
Supplement: Supplementary file 1 — Supplementary Material [file PRP2-8-e00586-s001.docx]

**Supporting Information**

Structurally Unique PARP-1 Inhibitors for the Treatment of Prostate Cancer

Ali Divan^1^, Alexei Tulin*^1^

^1^ University of North Dakota, School of Medicine & Health Sciences, Grand Forks, ND 58202

**Table of contents**

Supplemental Figure 1 ------------------------------------------------------------------------------------- S2

Supplemental Figure 2 ------------------------------------------------------------------------------------- S3

Supplemental Figure 3 ------------------------------------------------------------------------------------- S4

Supplemental Figure 4 ------------------------------------------------------------------------------------- S5

Supplemental Figure 5 ------------------------------------------------------------------------------------- S6

Supplemental Table 1 -------------------------------------------------------------------------------------- S7

**
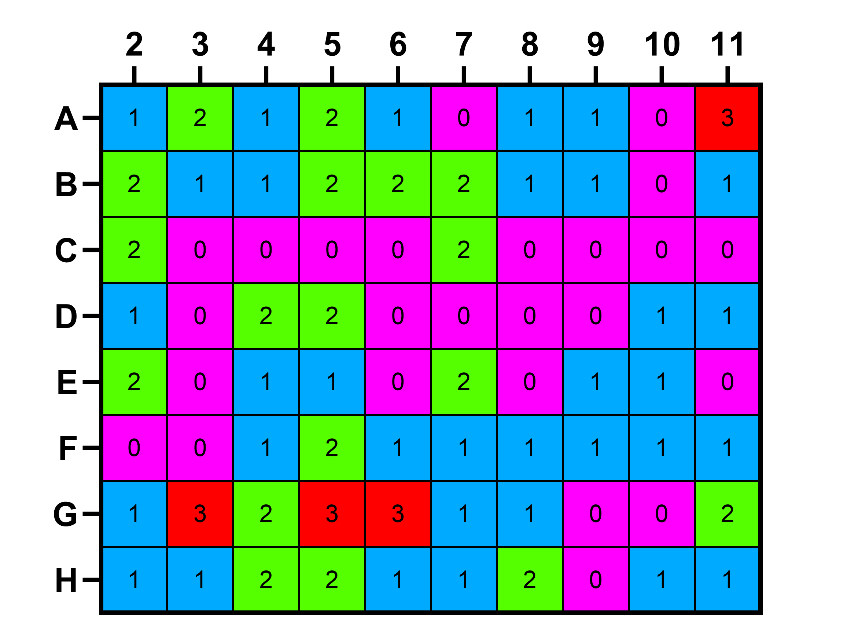
**

**Supplemental Figure 1**. Hit distribution map of all 65 positive hits from 8x 96 well plates totaling 664 tested compounds. Columns 1 and 12 contained positive controls and were thus omitted from the hit map. All values were combined to a 2x2 table with columns (2-6, 7-11) and rows (A-D, E-H), and Fisher’s exact test was performed. p> 0.9999 failed to reject null hypothesis that the hit distribution was a constant surface.

**
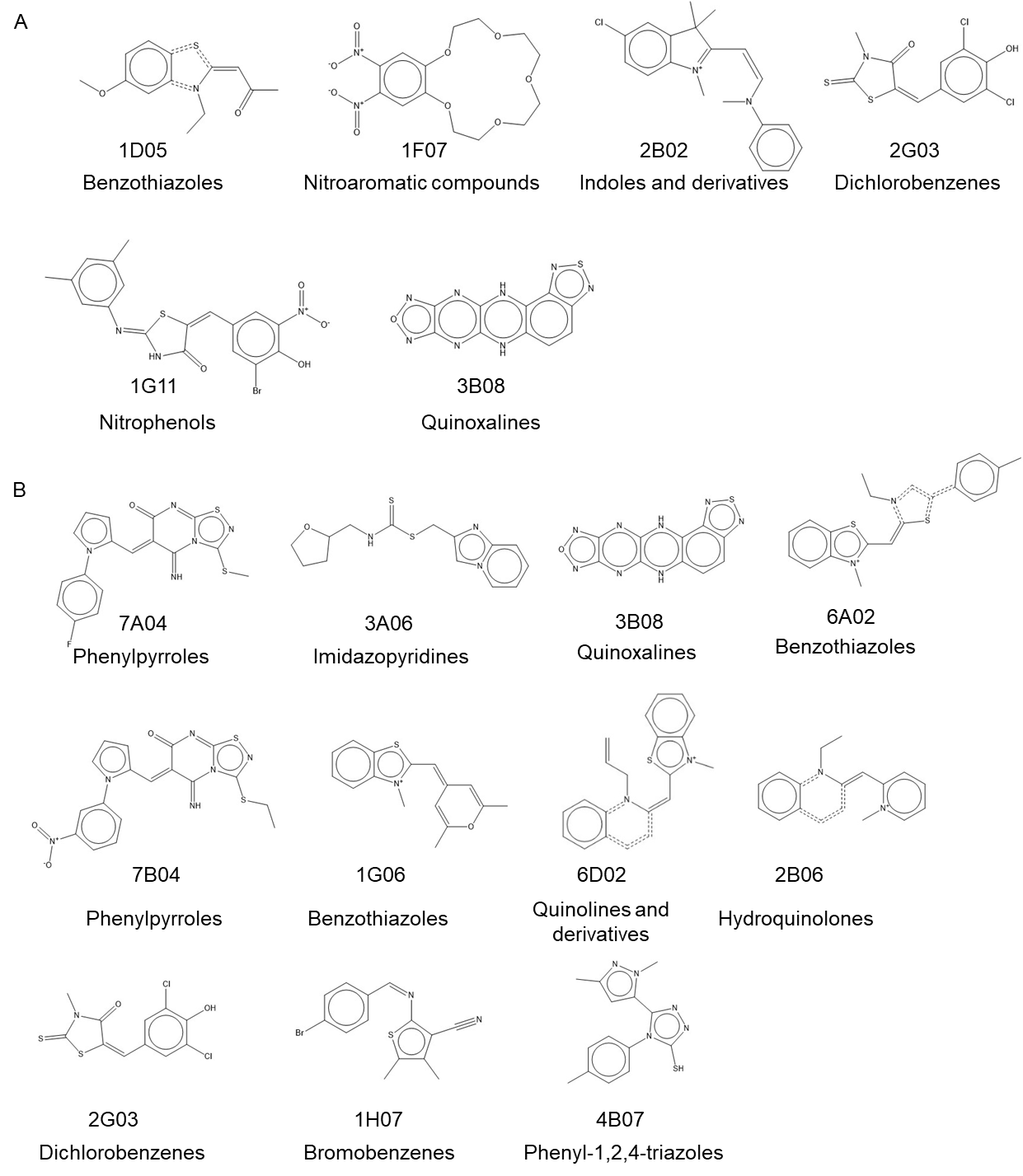
**

**Supplemental Figure 2. A)** Chemical structures and taxonomic classification of 6 molecules described in Fig 3B. **B)** Chemical structures and taxonomic classification of 11 molecules described in Fig 3D.


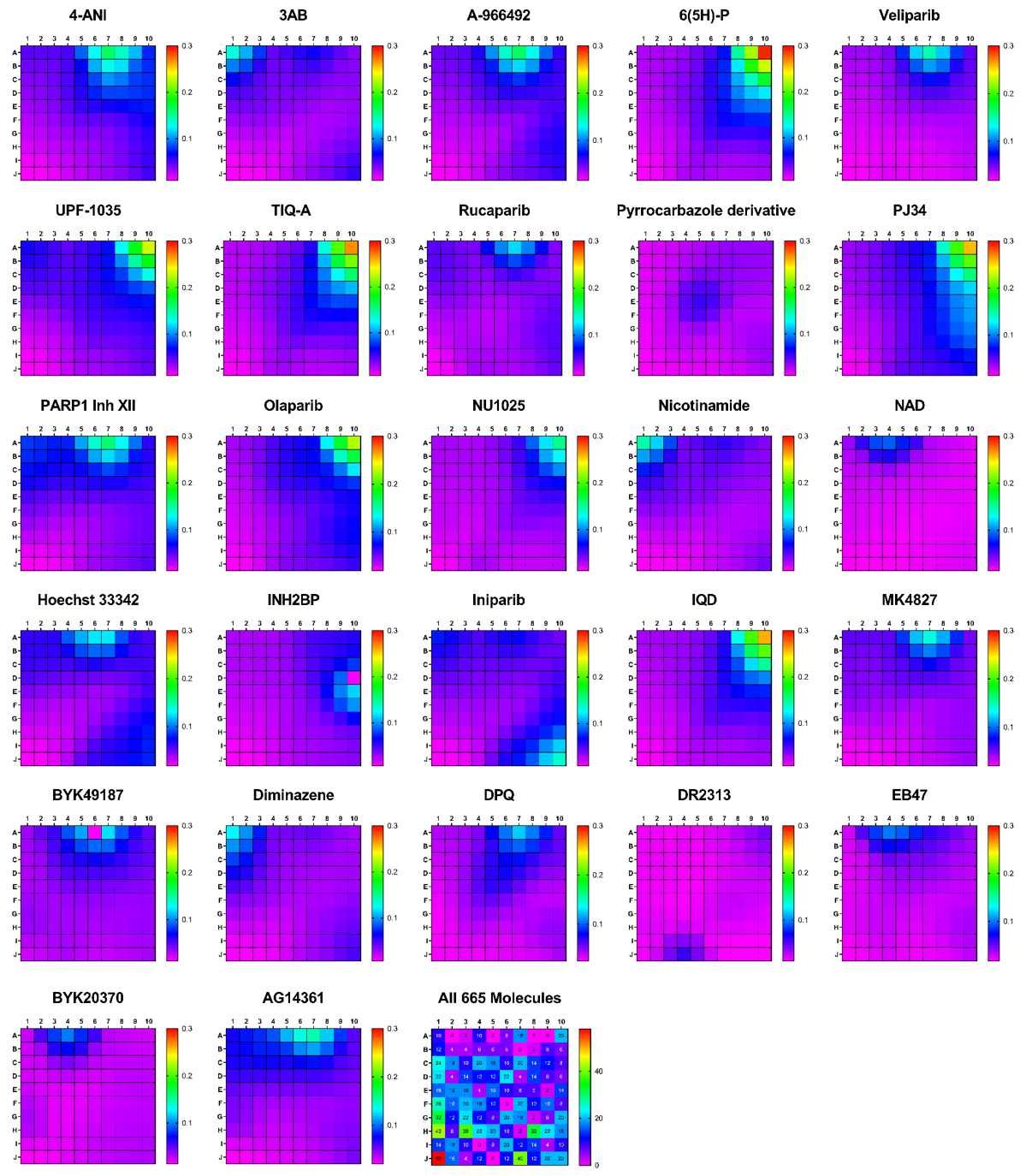


**Supplemental Figure 3**. Tanimoto similarity scores for each known PARP-1 inhibitor compared to respective 3D bin. Locations of all 665 small molecules and 3D isomers identified by cell-free assay and number of molecules per bin are shown in last heat map.


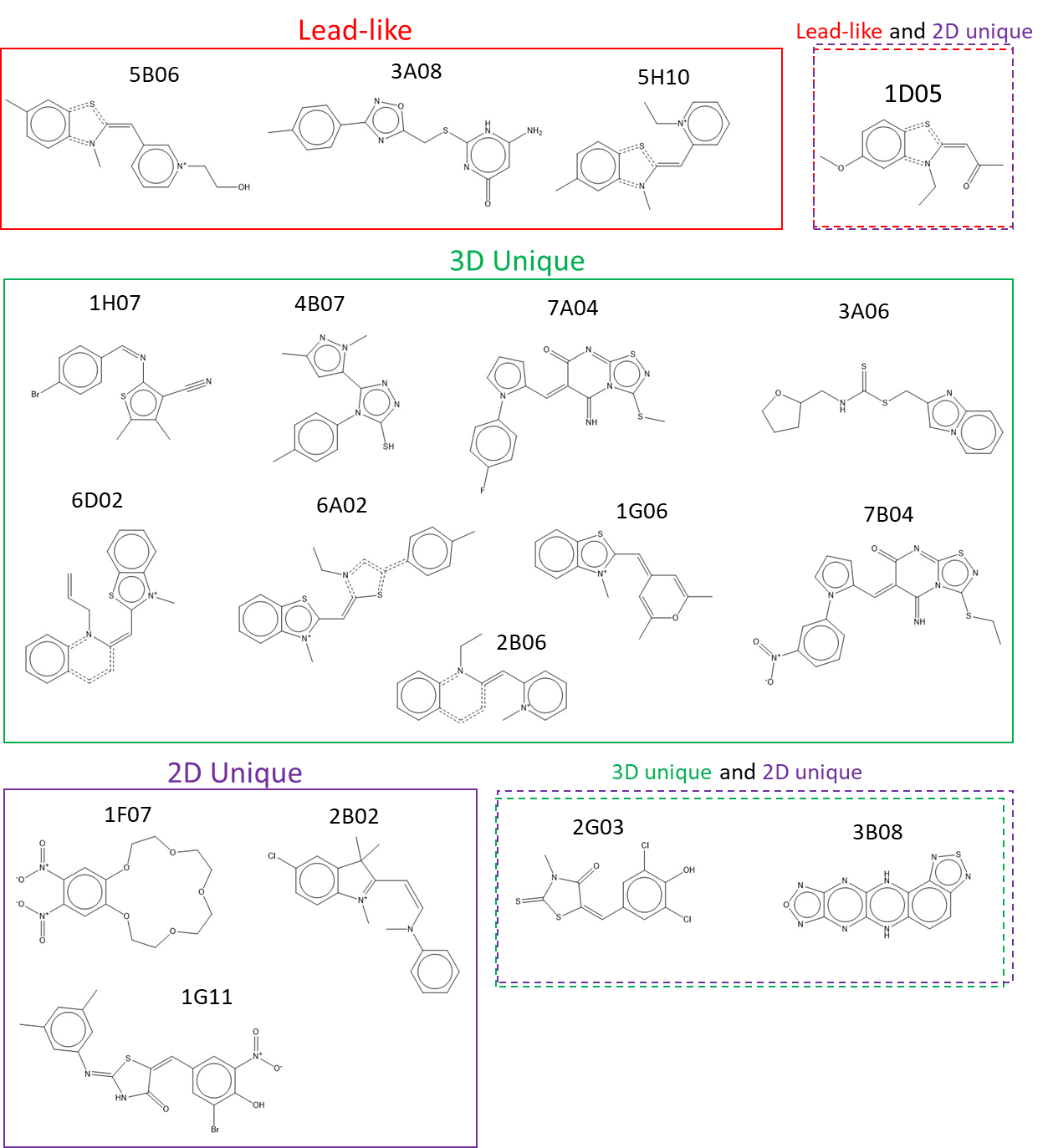


**Supplemental Figure 4.** Chemical structures of top hits identified by each method; Lead-like molecules (red box) were identified by SWISS-ADME (Fig 2A-E); 2D unique molecules (purple box) were identified by 2D Single linkage distance matrix hierarchical clustering (Fig 3A-B); 3D unique molecules (green box) were identified by 3D self-organizing maps calculated as the sum of fingerprint distances (Fig 3C-D); molecules identified as hits by more than one method are circumscribed by color-coded dashed lines.


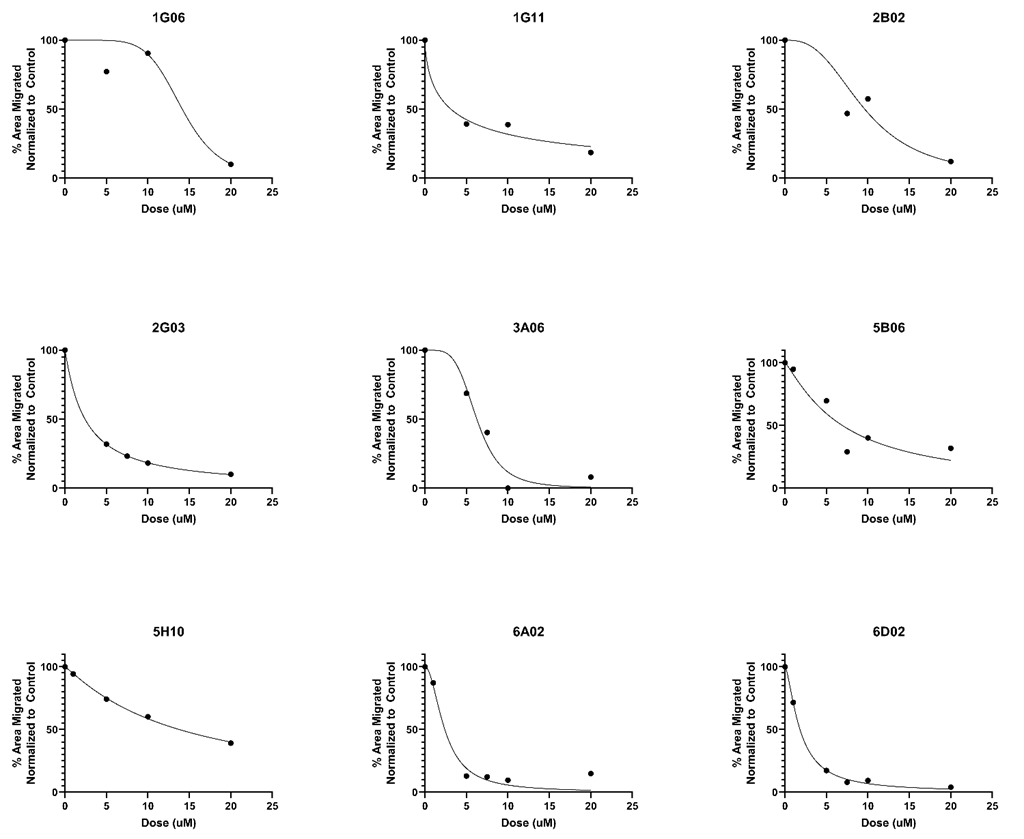


**Supplemental Figure 5.** Dose response data from top 9 molecules showing dose-response relationship. Data were obtained using migration assay. Curves were generated using Graphpad Prism software using the calculation of inhibitor vs normalized response, variable slope. Each data point represents a single dose tested in a 96-well plate.
