# Supplementary figures and images for: Structurally unique PARP‐1 inhibitors for the treatment of prostate cancer
Source: Pharmacol Res Perspect. 2020 Apr 28;8(2):e00586. doi: 10.1002/prp2.586 (PMC7186898; doi:10.1002/prp2.586)

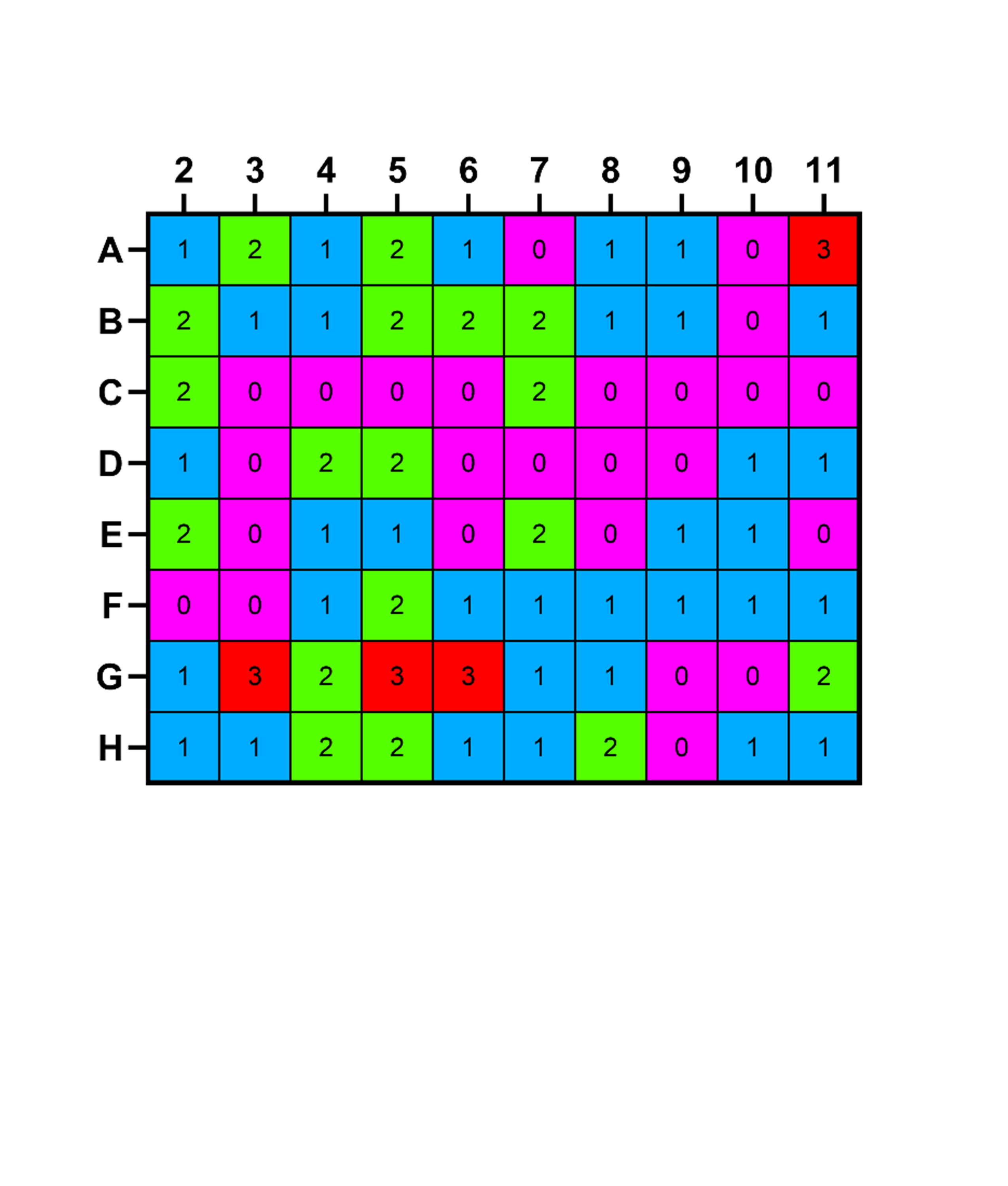

Supplement: Supplementary file 3 [file PRP2-8-e00586-s003.tif]

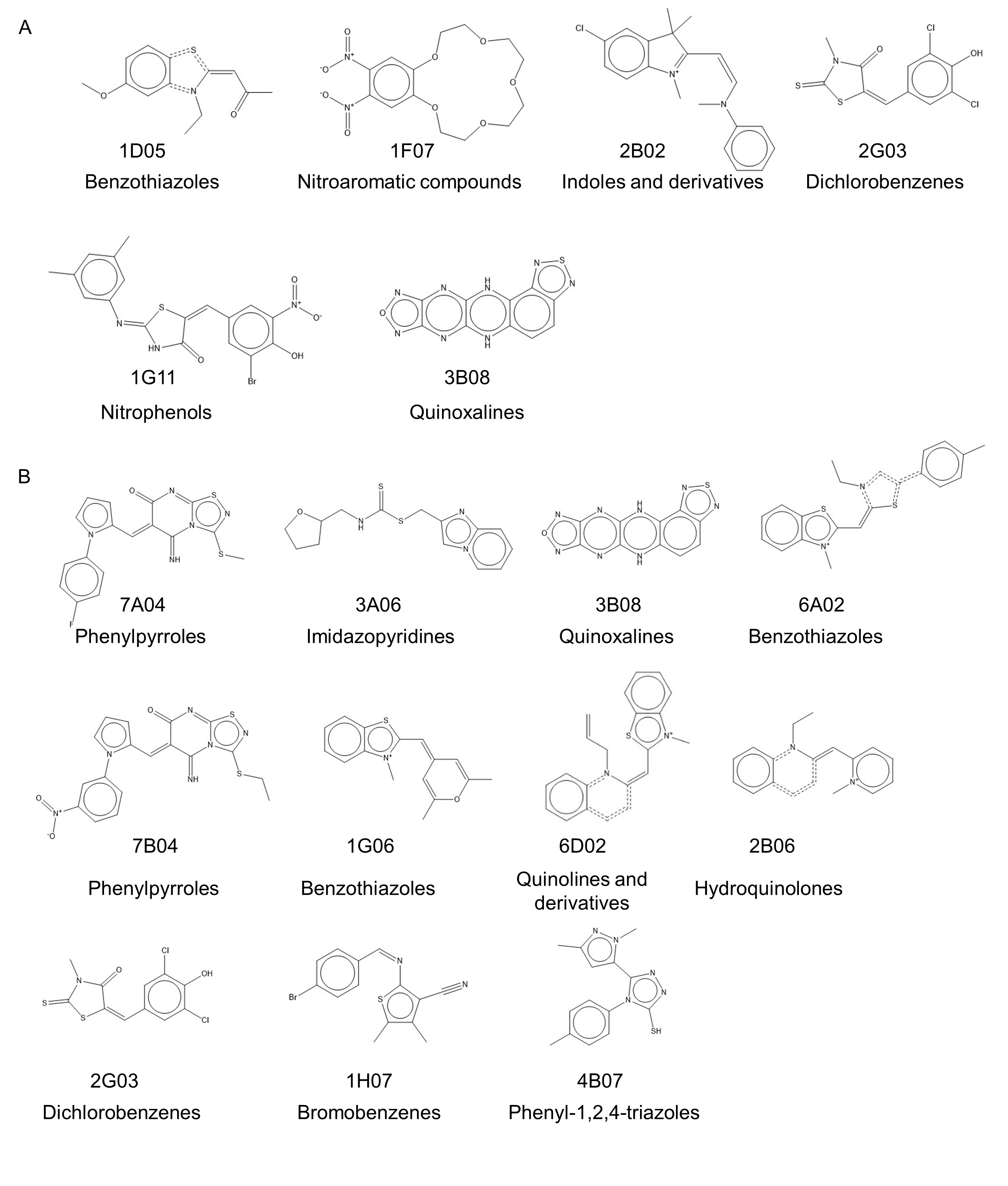

Supplement: Supplementary file 4 [file PRP2-8-e00586-s004.tif]

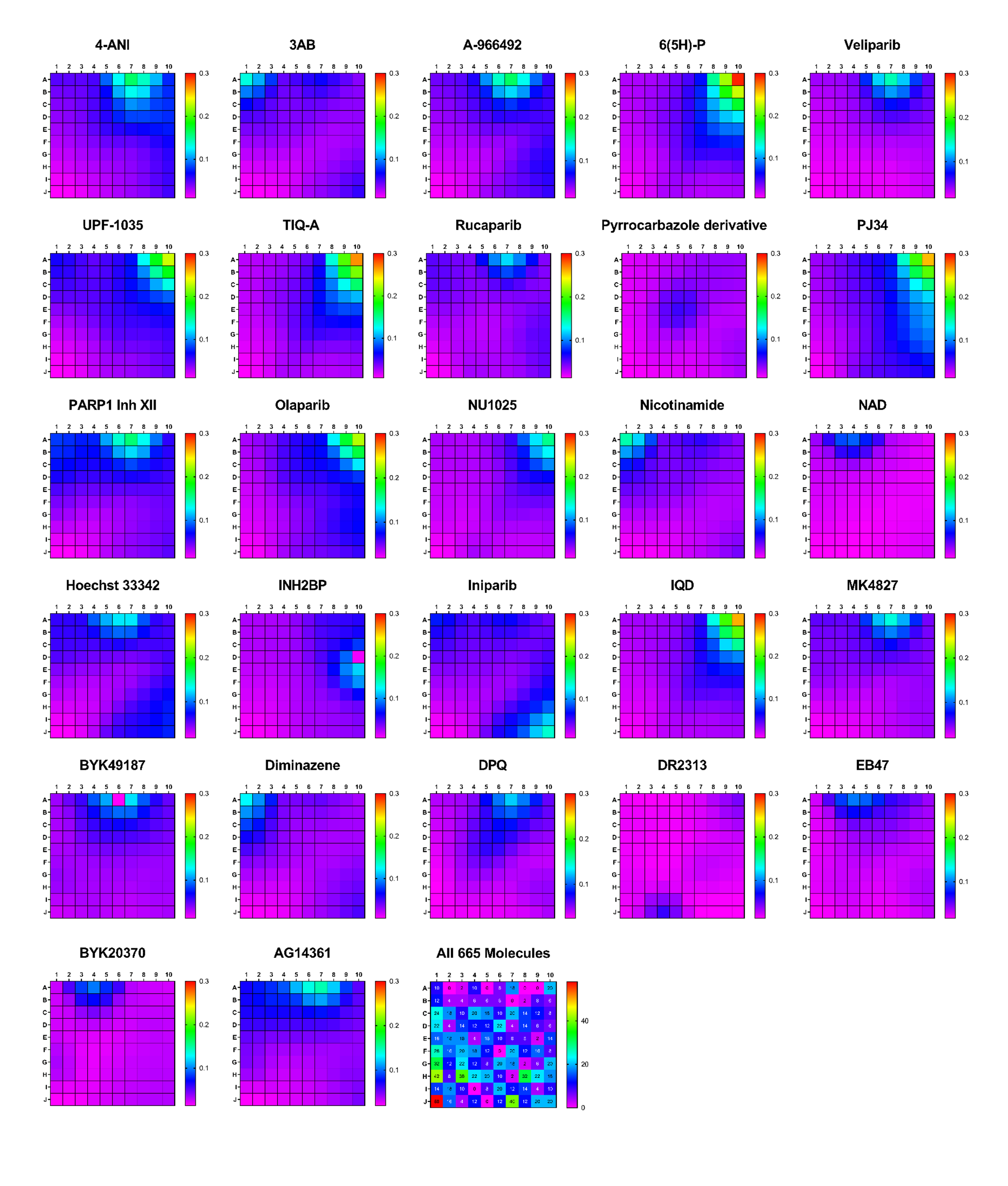

Supplement: Supplementary file 5 [file PRP2-8-e00586-s005.tif]

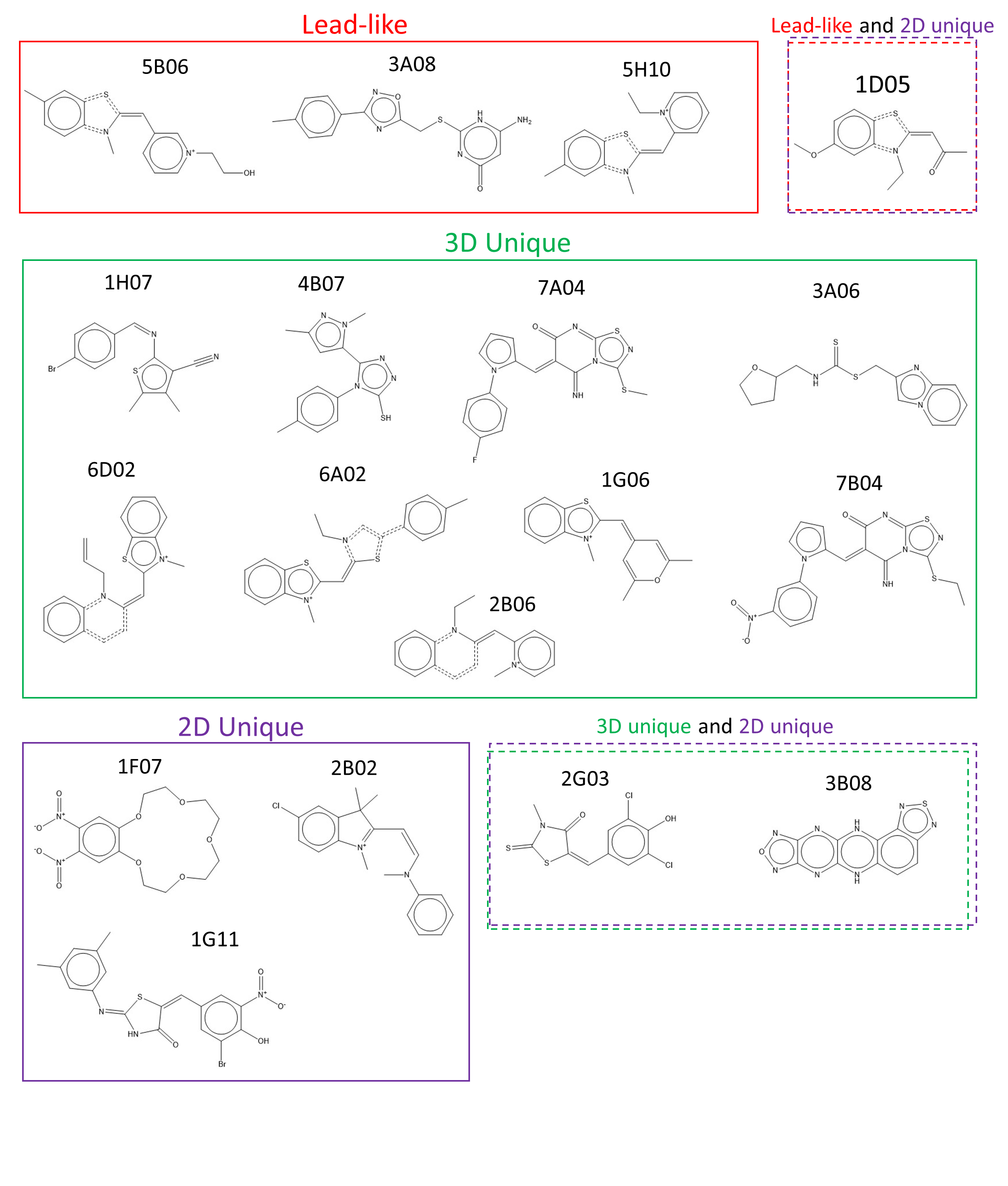

Supplement: Supplementary file 6 [file PRP2-8-e00586-s006.tif]

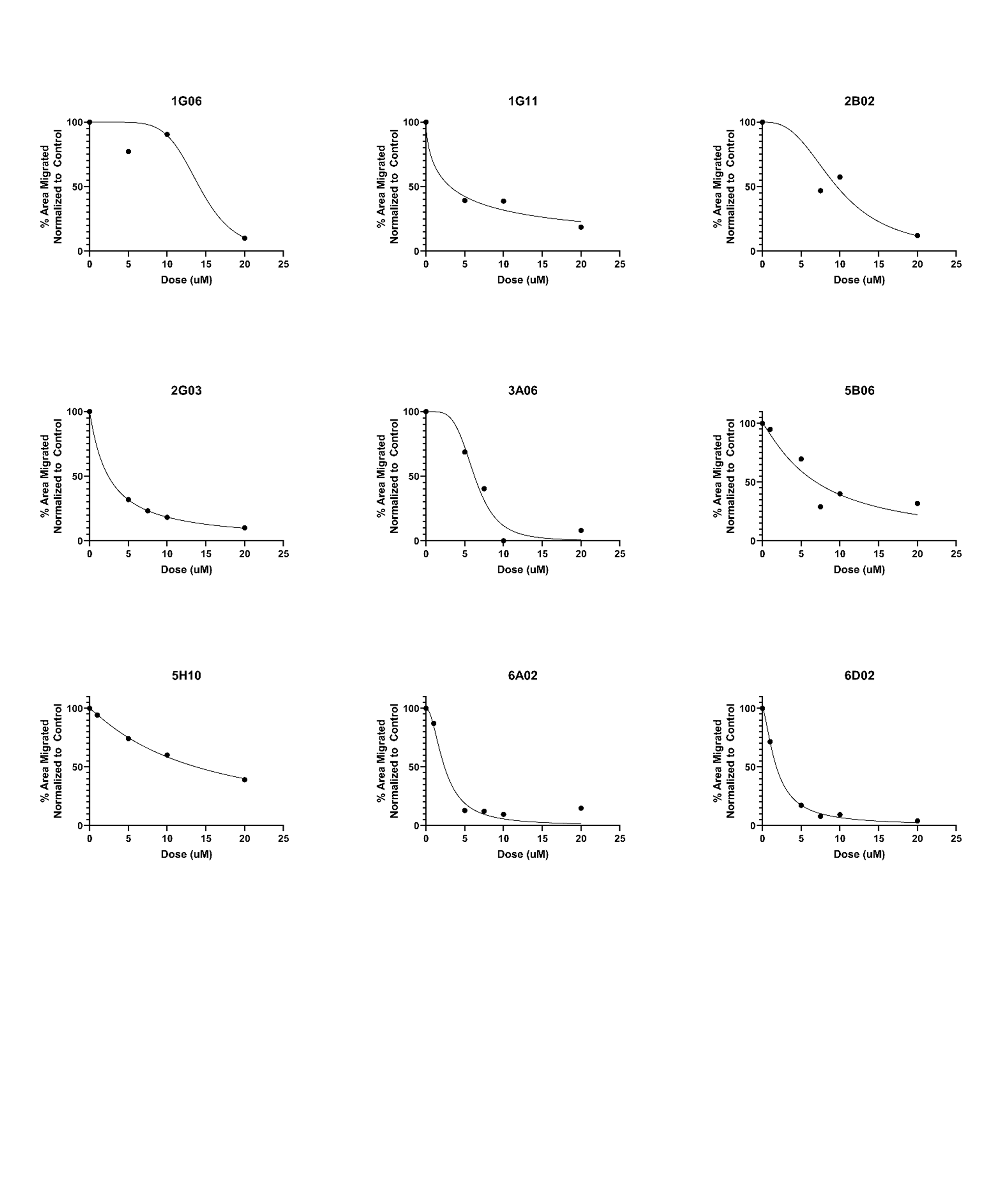

Supplement: Supplementary file 7 [file PRP2-8-e00586-s007.tif]
